# Metabolomic signatures mediate the association between physical frailty and metabolic dysfunction-associated steatotic liver disease: a prospective cohort study

**DOI:** 10.3389/fendo.2026.1845611

**Published:** 2026-05-12

**Authors:** Doudou Li, Yacong Bo, Yao Chen, Zhitian Guo, Li Li, Wenjie Zhang, Mingyi Xue, Yongjian Zhu, Liuhang Ren, Tingting Li, Zhan Gao

**Affiliations:** 1 The Fifth Affiliated Hospital of Zhengzhou University, Zhengzhou, China; 2 School of Public Health, Zhengzhou University, Zhengzhou, China; 3 National Health Commission (NHC) Key Laboratory of Birth Defects Prevention & Institute of Reproductive Health, Henan Academy of Innovations in Medical Science, Zhengzhou, China; 4 Department of Cardiology, The First Affiliated Hospital of Zhengzhou University, Zhengzhou, China

**Keywords:** mediation, metabolic dysfunction-associated steatotic liver disease, metabolic signature, physical frailty, UK Biobank

## Abstract

**Background:**

Physical frailty is linked to metabolic dysfunction-associated steatotic liver disease (MASLD), but the underlying metabolic mechanisms remain unclear. This study aimed to identify a frailty-related metabolic signature and examine its association with incident MASLD and its mediating role in the frailty-MASLD relationship.

**Methods:**

We analysed data from 244,187 UK Biobank participants. Frailty was assessed using the Fried Frailty Phenotype. Incident MASLD was ascertained via hospital records and death registries. An elastic net regression model identified frailty-associated metabolites to construct a weighted metabolic signature. Cox proportional hazards models estimated associations with MASLD risk, and mediation analysis quantified the signature’s contribution.

**Results:**

Over a median follow-up of 13.7 years, 3,408 incident MASLD cases occurred. A 96-metabolite signature was identified. Each 1-standard deviation increase in the signature was associated with a 21% higher MASLD risk (HR = 1.21, 95% CI: 1.16-1.25). Compared with robust participants, pre-frail and frail individuals had HRs of 1.51 (95% CI: 1.40-1.63) and 2.22 (95% CI: 1.97-2.50), respectively. The metabolic signature mediated 4.25% of the frailty-MASLD association.

**Conclusion:**

Frailty and its associated metabolic signature are independently associated with increased incident MASLD risk. The signature partially mediates this relationship, suggesting metabolic dysregulation links physical frailty to hepatic steatosis. Identifying this signature may enable earlier MASLD detection in frail individuals.

## 1 Introduction

Metabolic dysfunction-associated steatotic liver disease (MASLD), formerly known as non-alcoholic fatty liver disease, represents the most prevalent chronic liver condition globally, affecting approximately 38% of adults worldwide with rising incidence parallel to the obesity and diabetes pandemics (1–3). As the hepatic manifestation of systemic metabolic dysregulation, MASLD is characterized by excessive hepatic fat accumulation (≥5%) in the absence of excessive alcohol consumption or alternative aetiologies (4). With MASLD now ranking as the second leading cause of end-stage liver disease and liver transplantation in developed nations, and economic projections exceeding $1 trillion by 2030 (3, 5), the pathogenesis of this condition has become a critical research priority. Its pathogenesis centres on multifactorial metabolic perturbations, including insulin resistance, adipose tissue lipotoxicity, and chronic low-grade inflammation (6, 7). Yet, modifiable risk factors beyond traditional metabolic syndrome components remain incompletely characterized, and the mechanisms driving disease progression from simple steatosis to advanced fibrosis and hepatocellular carcinoma are not fully elucidated (7). Identifying novel risk factors and their metabolic mediators is therefore imperative for developing targeted prevention strategies and enabling earlier detection of high-risk individuals.

Beyond traditional metabolic risk factors, physical frailty has emerged as a novel determinant of metabolic health across the lifespan. Frailty is a complex clinical condition characterized by a decline in physiological capacity across multiple organ systems, leading to increased vulnerability to external stressors (8). While classically considered a geriatric syndrome, recent evidence highlights its prevalence and clinical relevance in middle-aged and even younger adults (9). Notably, metabolic alterations characterizing frailty, including dysregulated amino acid profiles (e.g., elevated glutamate), perturbed lipid metabolism (e.g., altered VLDL and fatty acid composition), and altered hormonal axes, mirror established pathways in MASLD pathogenesis (10–12). This metabolic convergence suggests that a frailty-associated metabolic signature may serve as a mechanistic intermediary linking physical frailty to hepatic steatosis. However, whether this signature prospectively predicts incident MASLD and quantitatively mediates the frailty-MASLD association remains to be elucidated.

To elucidate these metabolic pathways, metabolic signatures, defined as composite profiles of multiple metabolites reflecting integrated metabolic states, provide a powerful approach to capture the complex network perturbations underlying both conditions (13, 14). While previous studies have employed metabolic signatures to predict diabetes, cardiovascular disease, and mortality (14, 15), no study has derived a frailty-specific metabolic signature or examined its longitudinal association with incident MASLD. This gap is particularly significant given that identifying such a signature could elucidate the metabolic pathways linking frailty to hepatic steatosis and enable earlier detection of high-risk individuals. We hypothesized that the frailty-related metabolic signature would partially mediate the association between physical frailty and MASLD risk. Therefore, leveraging the large-scale prospective UK Biobank cohort, this study aimed to: (1) identify a metabolic signature associated with physical frailty; (2) examine its prospective association with incident MASLD risk; and (3) quantify its mediating role in the frailty-MASLD relationship.

## 2 Methods

### 2.1 Participants

This study used data from the UK Biobank, a prospective cohort study that recruited 502,366 adults aged 37–73 years from 22 assessment centres across England, Scotland, and Wales between 2006 and 2010 (16). The study population initially comprised 502,366 participants. We excluded 570 participants with prevalent MASLD at baseline, 12,672 participants with missing frailty data, and 244,937 participants without metabolomic data. Ultimately, 244,187 participants were included in the final analysis (**Supplementary Figure 1**). The UK Biobank study was approved by the National Information Governance Board for Health and Social Care and the NHS North West Multicentre Research Ethics Committee, and all participants provided written informed consent. This research was conducted using the UK Biobank resource under application number 93398.

### 2.2 Assessment of physical frailty

Physical frailty was assessed using the Fried Frailty Phenotype, which includes five criteria: unintentional weight loss, exhaustion, low physical activity, slow gait speed, and weakness (low grip strength) (17). These indicators were adapted for use in the UK Biobank cohort using available proxy measures (9, 17, 18). Detailed definitions of these indicators and corresponding UK Biobank Field IDs are provided in **Supplementary Table 1**. Briefly, each criterion was scored as 0 (absent) or 1 (present), yielding a total score ranging from 0 to 5. Participants were subsequently categorized as robust (0 points), pre-frail (1–2 points), or frail (≥3 points) (17).

### 2.3 Plasma metabolite profiling

Plasma metabolites were quantified using a high-throughput NMR-based metabolic biomarker profiling platform (Nightingale Health, Helsinki, Finland) in approximately 280,000 participants between June 2019 and April 2020 (19, 20). This study included 251 metabolic biomarkers, encompassing low-molecular-weight metabolites (e.g., amino acids, ketone bodies, glycolysis-related metabolites), lipoprotein lipids across 14 subclasses, apolipoproteins, fatty acids, and inflammatory markers. The profiling process involved quality control to eliminate systematic and technical variability, with detailed information published online (21). For the analysis, each metabolite underwent natural logarithm transformation to normalize the data distribution.

### 2.4 Outcome ascertainment

Diagnostic information was obtained from hospital inpatient records (Hospital Episode Statistics) and death registries. Incident MASLD was defined as a primary or secondary diagnosis of ICD-10 code K76.0 in hospital admission records, combined with the presence of at least one cardiometabolic risk factor (overweight/obesity, type 2 diabetes mellitus or impaired glucose regulation, hypertension, hypertriglyceridemia, or low HDL-cholesterol) (22). Detailed definitions of these risk factors and corresponding UK Biobank Field IDs are provided in **Supplementary Table 2**. Hospital admissions data were available until October 2022 in England, August 2022 in Scotland, and May 2022 in Wales. Participants were followed up from baseline (2006–2010) until the earliest of: date of first MASLD diagnosis, date of death, date of loss to follow-up, or end of follow-up (October 2022 in England, August 2022 in Scotland, and May 2022 in Wales).

### 2.5 Covariates

Sociodemographic factors (age, sex, ethnicity, Townsend deprivation index, educational qualifications), lifestyle factors (smoking status, alcohol consumption, diet quality score), and components of metabolic syndrome (central obesity, hypertension, hyperglycaemia/diabetes, high triglycerides, and low HDL cholesterol) were collected at baseline. Age and sex were self-reported. Townsend deprivation index was derived from participants’ postcode of residence using the Townsend score (higher scores indicating greater socioeconomic deprivation). Educational qualifications were categorized as university/college degree and below university level. Smoking status and alcohol consumption were self-reported and categorized as never, former, or current. Diet quality score was calculated based on seven components (higher intake of fruits, vegetables, fish, whole grains; lower intake of processed meats, unprocessed red meats, refined grains) and categorized as unhealthy (score <4) or healthy (score ≥4) (23). Central obesity was characterized by a waist circumference exceeding 88cm in women or 102cm in men (24). Hyperglycaemia/diabetes was identified by a fasting glucose level surpassing 5.6 mmol/l or self-reported physician diagnosis of diabetes (24). Hypertension was defined as systolic blood pressure ≥130 mmHg and/or diastolic blood pressure ≥85 mmHg, or self-reported physician diagnosis of hypertension (24). High triglycerides were defined as fasting triglycerides ≥1.7 mmol/L (25). Low HDL cholesterol was defined as <1.0 mmol/L in men and <1.3 mmol/L in women (25). Physical activity was not treated as a confounder because it is one of the components of frailty.

### 2.6 Statistical analyses

#### 2.6.1 Identification of metabolic signature for frailty

The metabolic signature of frailty was constructed using metabolomic profiles from the UK Biobank. Elastic net regression was used to identify metabolites associated with frailty, with L1 (lasso) and L2 (ridge) penalty terms to handle high-dimensional data (14). Prior to analysis, all metabolites were standardized to z-scores (mean = 0, SD = 1). The regularization parameter λ was selected via 10-fold cross-validation using the cv.glmnet function (14). We selected lambda.1se (the largest λ within 1 standard error of the minimum MSE) to obtain a parsimonious model. Final model coefficients (representing metabolite weights) were obtained using the glmnet function at the selected λ value (14). The metabolic signature was calculated as the weighted sum of metabolites with non-zero coefficients, standardized to z-scores, with higher scores indicating a metabolic profile associated with frailty.

#### 2.6.2 Associations between frailty, metabolic signature, and MASLD risk

To assess the associations of frailty and metabolic signature with incident MASLD, Cox proportional hazards models were employed to estimate hazard ratios (HRs) and 95% confidence intervals (CIs), after confirming the proportional hazards assumption using Schoenfeld residuals. Three modelling approaches were used: (1) Age- and sex-adjusted models (Model 1); (2) Multivariable-adjusted models additionally adjusting for socioeconomic factors (Townsend deprivation index, ethnicity, education), lifestyle factors (smoking, alcohol consumption, diet quality score), and metabolic factors (central obesity, hypertension, hyperglycaemia/diabetes, high triglycerides, low HDL cholesterol) (Model 2); and (3) Mutually adjusted models including both frailty and metabolic signature simultaneously to assess their independent effects (Model 3). The dose-response relationship between cumulative number of frailty components and MASLD was assessed using a restricted cubic splines model. Associations of individual frailty components with MASLD were examined, adjusting for Model 2 covariates and mutually for other four components to identify component-specific effects. Bonferroni correction was applied for multiple testing in these analyses.

#### 2.6.3 Subgroup and sensitivity analysis

To assess whether the association between frailty and MASLD differed by subgroups, we tested for multiplicative interactions and stratified analyses by age, sex, smoking status, and obesity status. Several sensitivity analyses were conducted to examine the robustness of the primary findings, including: 1) excluding participants who developed incident MASLD within the first 2 years of follow-up to address reverse causation; 2) excluding participants with prevalent cardiovascular disease or cancer at baseline to minimize the effects of comorbidities; 3) using weekly alcohol consumption (continuous) instead of categorical alcohol consumption status; and 4) complete-case analysis.

#### 2.6.4 Mediation analysis

Mediation analysis was conducted to quantify the mediating role of the metabolic signature in the association between frailty and MASLD, as well as for individual frailty components. The total effect was decomposed into natural direct effects (NDE), natural indirect effects (NIE) (26). In the mediation analysis, frailty and its components were defined as the independent variable, MASLD as the dependent variable, and the metabolic signature as the mediator, adjusting for covariates as in the main multivariable model. We used 5000 bootstrap resamples to estimate 95% confidence intervals for the mediation effects (27). Causal mediation analysis was conducted using the R package CMAverse (version 0.1.0) (27).

All statistical tests were two-sided, and P < 0.05 was considered statistically significant, unless otherwise specified. All analyses were conducted using R version 4.3.2 (R Foundation for Statistical Computing, Vienna, Austria).

## 3 Results

### 3.1 Participant characteristics

A total of 244,187 participants were included in the final analysis. At baseline, participants had a mean (SD) age of 57.1 (8.07) years, and 52.9% were female. Overall, 126,115 (51.6%), 106,873 (43.8%), and 11,199 (4.6%) participants were classified as robust, pre-frail, and frail, respectively. The median (IQR) follow-up was 13.7 (12.9-14.5) years, during which 3,408 participants developed MASLD. Compared with robust participants, those who were pre-frail or frail were more likely to be older, socioeconomically deprived, less educated, current smokers, non-drinkers, and have unhealthy dietary patterns. Additionally, pre-frail and frail participants had higher prevalences of central obesity, hypertension, hyperglycaemia/diabetes, low HDL cholesterol, and high triglycerides (**Table 1**).

**Table 1:** Characteristics of the population by frailty phenotype.

Characteristics	Total (n =244,187)	Robust (n =126,115)	Pre-frail (n =106,873)	Frail (n =11,199)
**Age**	57.10 (8.07)	56.57 (8.06)	57.58 (8.09)	58.60 (7.69)
Sex, n (%)				
Female	129,260 (52.9)	64,653 (51.3)	58,194 (54.5)	6,413 (57.3)
Male	114,927 (47.1)	61,462 (48.7)	48,679 (45.5)	4,786 (42.7)
Education Level, n (%)				
University/College	72,936 (29.9)	31,990 (25.4)	35,270 (33.0)	5,676 (50.7)
Others	171,251 (70.1)	94,125 (74.6)	71,603 (67.0)	5,523 (49.3)
BMI, n (%)				
Normal	77,723 (31.8)	44,129 (35.0)	31,281 (29.3)	2,313 (20.7)
Over weight	105,594 (43.2)	56,545 (44.8)	45,307 (42.4)	3,742 (33.4)
Obese	60,870 (24.9)	25,441 (20.2)	30,285 (28.3)	5,144 (45.9)
Ethnicity, n (%)				
White	232,183 (95.1)	121,896 (96.7)	100,278 (93.8)	10,009 (89.4)
Others	12,004 (4.9)	4,219 (3.3)	6,595 (6.2)	1,190 (10.6)
**TDI**	-1.39 (3.05)	-1.77 (2.83)	-1.13 (3.15)	0.38 (3.55)
Smoke status, n (%)				
Never	133,571 (54.7)	71,595 (56.8)	56,988 (53.3)	4,988 (44.5)
Previous	85,023 (34.8)	43,682 (34.6)	37,387 (35.0)	3,954 (35.3)
Current	25,593 (10.5)	10,838 (8.6)	12,498 (11.7)	2,257 (20.2)
Drink status, n (%)				
Never	10,488 (4.3)	3,802 (3.0)	5,536 (5.2)	1,150 (10.3)
Previous	8,671 (3.6)	2,969 (2.4)	4,508 (4.2)	1,194 (10.7)
Current	225,028 (92.2)	119,344 (94.6)	96,829 (90.6)	8,855 (79.1)
**Diet pattern (healthy), n (%)**	144,178 (59.0)	77,746 (61.6)	61,243 (57.3)	5,189 (46.3)
Metabolic syndrome, n (%)				
Central obesity (yes)	76,318 (31.3)	32,560 (25.8)	37,670 (35.2)	6,088 (54.4)
High blood pressure/hypertension (yes)	164,425 (67.3)	85,506 (67.8)	71,443 (66.8)	7,476 (66.8)
High triglycerides (yes)	101,943 (41.7)	50,347 (39.9)	45,870 (42.9)	5,726 (51.1)
Low HDL (yes)	51,262 (21.0)	21,974 (17.4)	25,222 (23.6)	4,066 (36.3)
High glycaemia/diabetes (yes)	42,063 (17.2)	18,142 (14.4)	20,417 (19.1)	3,504 (31.3)

Data are presented as mean ± SD or n (%). TDI, Townsend deprivation index; BMI, body mass index.

### 3.2 Variation of the metabolites in response to frailty

**Figure 1:**
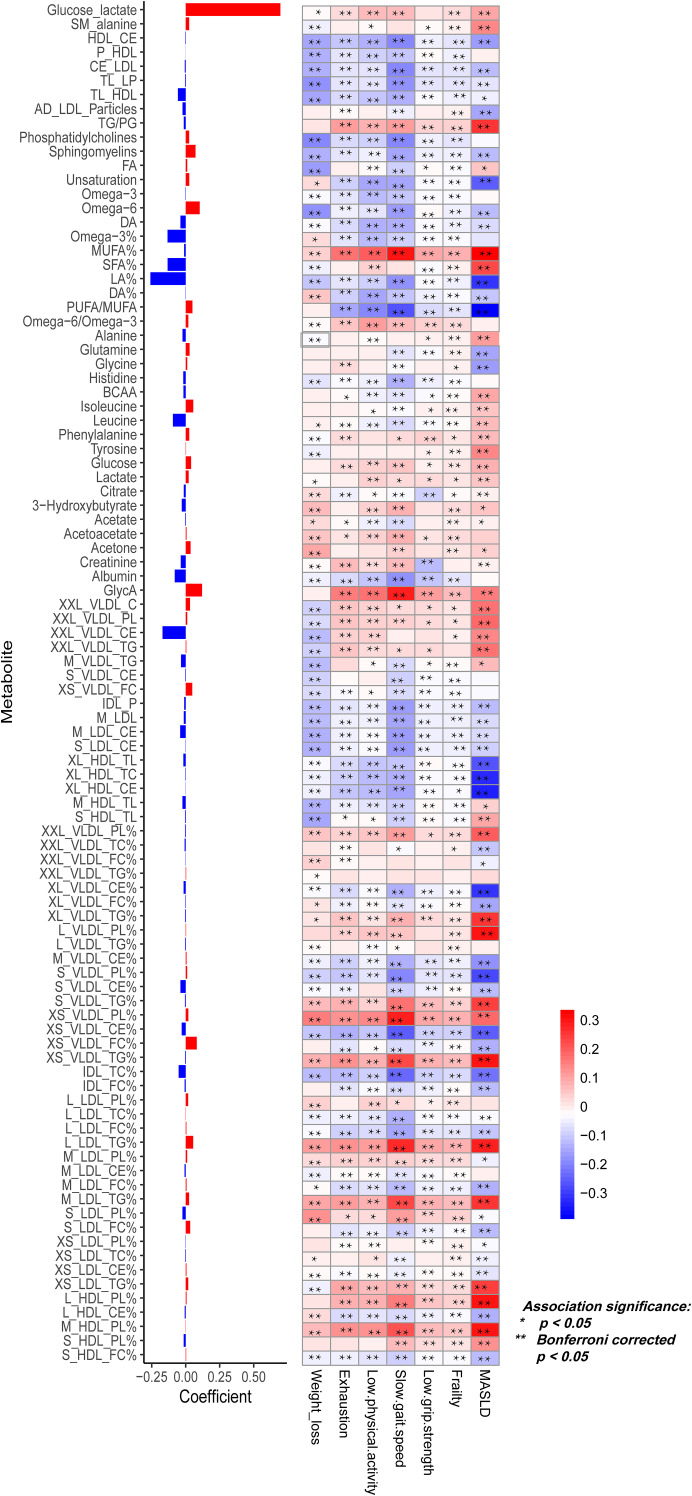
Associations of the 96 - metabolite metabolic signature with frailty and MASLD risk. Presented from left to right are the coefficients (weights) of metabolites within the signature and the associations of each metabolite with frailty components, overall frailty status, and subsequent MASLD risk via a heatmap, where colours reflect the direction (red-positive and blue-inverse) and magnitude of associations, and asterisks indicate statistical significance (*P < 0.05 and **Bonferroni-corrected P < 0.05). AD, average diameter; CE, cholesteryl ester; CI, confidence interval; DA, docosahexaenoic acid; FA, total fatty acids; FC, free cholesterol; GlycA, glycoprotein -acetyls; HDL, high-density lipoprotein; HR, hazard ratio;IDL, intermediate density lipoprotein; L, large; LA, linoleic acid; LDL, low-density lipoprotein; LP, lipoprotein particles; M, medium; MUFA, monounsaturated fatty acids; PG, phosphoglycerides; PL, phospholipid; PUFA, polyunsaturated fatty acids; S, small; SFA, saturated fatty acids; SM, Spectrometer; TC, total cholesterol; TL, total lipids; TP, total phospholipids; VLDL, very low-density lipoprotein; XL, very large; XS, very small; XXL, especially large.

**Figure 2:**
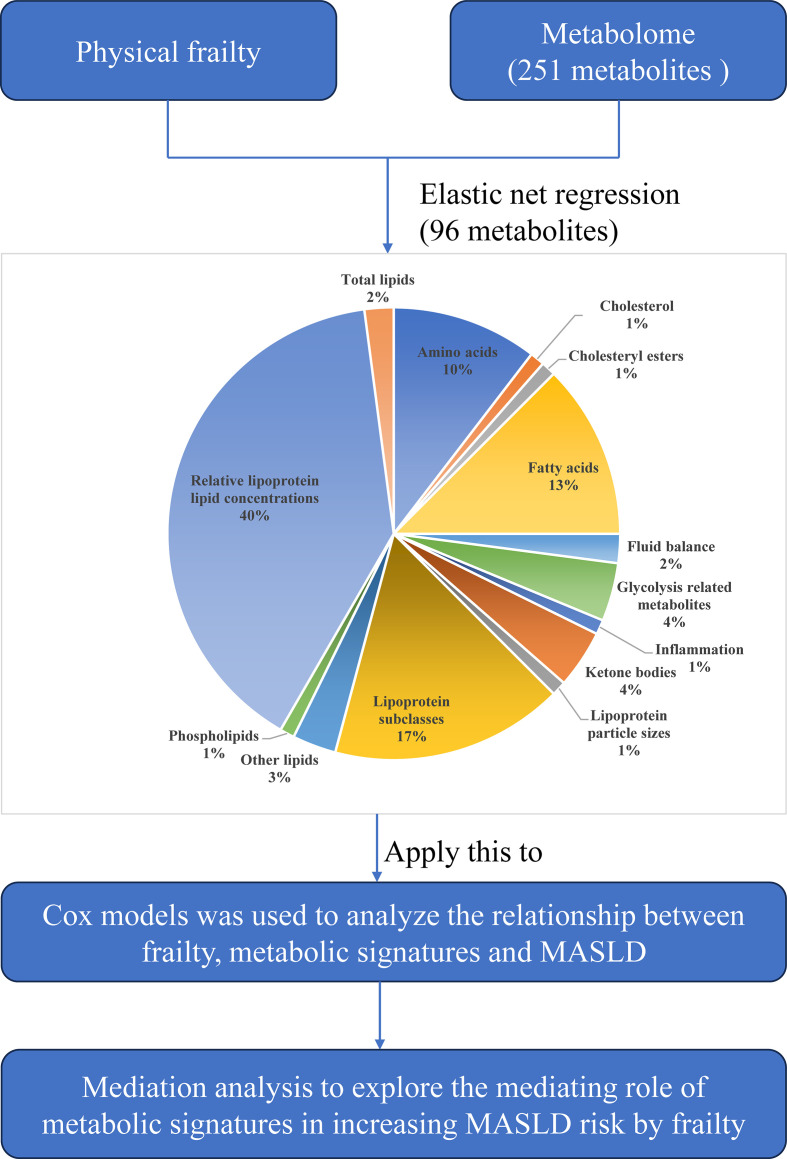
The metabolic signature for frailty flow chart for analytic approach validation. MASLD, metabolic dysfunction-associated steatotic liver disease.

Of the 251 metabolites, 96 (38.2%; 42 positively and 54 negatively associated) were significantly associated with frailty (**Figure 1**). The metabolic signature encompassed diverse metabolic classes, including lipoprotein particles and their lipid compositions, fatty acids, amino acids, ketone bodies, and metabolites related to glycolysis, inflammation, and fluid balance (**Figure 2**). Among the metabolites constituting the metabolic signature, the top five positive contributors were lactate, glycoprotein acetyls (GlycA), omega-6 fatty acid percentage (Omega-6%), free cholesterol to total lipids in very small VLDL percentage (XS_VLDL_FC%), and sphingomyelins to total phospholipids percentage. The top five negative contributors were linoleic acid percentage (LA%), cholesteryl esters in chylomicrons and extremely large VLDL (XXL_VLDL_CE), omega-3 fatty acid percentage (Omega-3%), saturated fatty acid percentage (SFA%), and leucine (**Figure 1**). These 96 metabolites were extensively associated with individual frailty components, including exhaustion, slow gait speed, and weakness (**Figure 1**).

We next examined whether these frailty-associated metabolites were prospectively associated with incident MASLD. Among the 96 metabolites, 30 were positively and 38 were negatively associated with MASLD, while 28 showed no significant association (**Supplementary Figure 2**). The top five positive associations with MASLD were monounsaturated fatty acid percentage (MUFA%), phospholipids to total lipids in large HDL percentage (L_HDL_PL%), triglycerides to total lipids in very small VLDL percentage (XS_VLDL_TG%), phospholipids to total lipids in medium HDL percentage (M_HDL_PL%), and phospholipids to total lipids in large VLDL percentage (L_VLDL_PL%). The top five negative associations were cholesteryl esters in extremely large VLDL percentage (XL_VLDL_CE%), linoleic acid percentage (LA%), total cholesterol in extremely large HDL (XL_HDL_TC), cholesteryl esters in extremely large HDL (XL_HDL_CE), and polyunsaturated to monounsaturated fatty acid ratio (PUFA/MUFA).

### 3.3 Association of frailty and the metabolic signature with MASLD

**Figure 3:**
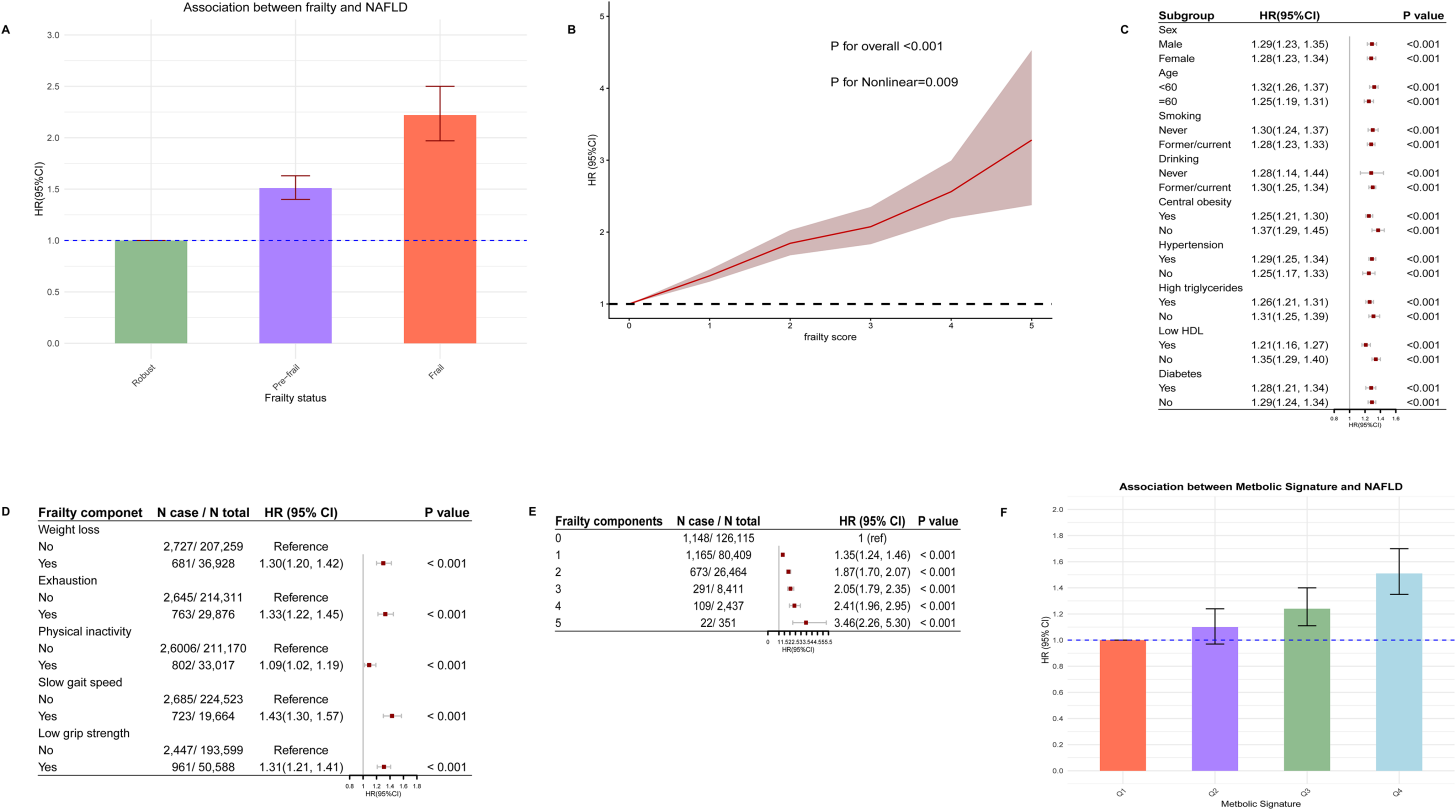
Association of Frailty and the Metabolic signature with MASLD. **(A)** Association between frailty and incident MASLD; **(B)** Non-linearity association of frailty with incident MASLD; **(C)** Association between frailty and incident MASLD in different population groups; **(D)** Associations between the frailty components and MASLD; **(E)** Association between the number of frailty components and incident MASLD; **(F)** Association between the metabolic signature and incident MASLD. MASLD, metabolic dysfunction-associated steatotic liver disease.

In Model 2, participants with pre-frail and frail status had higher risks of incident MASLD compared with robust participants, with HRs of 1.51 (95% CI: 1.40-1.63) and 2.22 (95% CI: 1.97-2.50), respectively (both *P* < 0.001) (**Figure 3A**; **Supplementary Table 3**). When analysed continuously, each one-point increase in the number of frailty components was associated with a 30% higher risk of incident MASLD (HR = 1.30, 95% CI: 1.26-1.34; *P* < 0.001) (**Supplementary Table 3**). We observed a non-linear association between frailty and incident MASLD (*P* for non-linearity = 0.009), with risk accelerating at higher frailty scores (**Figure 3B**). Subgroup analyses showed consistent associations across age, sex, and metabolic health subgroups (**Figure 3C**; **Supplementary Table 4**), and sensitivity analyses confirmed robustness after excluding early events and prevalent comorbidities (**Supplementary Tables 5**-**8**).

Additionally, unintentional weight loss (HR = 1.30, 95% CI: 1.20-1.42), exhaustion (HR = 1.33, 95% CI: 1.22-1.45), low physical activity (HR = 1.09, 95% CI: 1.02-1.19), slow gait speed (HR = 1.43, 95% CI: 1.30-1.57), and weakness (HR = 1.31, 95% CI: 1.21-1.41) were independently associated with MASLD risk after mutual adjustment (**Figure 3D**). The risk of incident MASLD also increased linearly with the number of frailty components (*P* for trend < 0.001) (**Figure 3E**).

In Model 2, participants in the highest quintile of the metabolic signature score had an increased risk of MASLD compared with those in the lowest quintile (HR = 1.51, 95% CI: 1.35-1.70; *P* < 0.001) (**Figure 3F**, **Supplementary Table 3**). When analysed continuously, the metabolic signature was significantly associated with MASLD risk (HR = 1.21, 95% CI: 1.16-1.25 per 1-SD increase; *P* < 0.001 (**Supplementary Table 3**).

To assess their independent effects, we mutually adjusted frailty and the metabolic signature in Model 3. After adjustment for the metabolic signature, frailty remained significantly associated with MASLD risk. Participants with pre-frail and frail status had HRs of 1.49 (95% CI: 1.38-1.61) and 2.13 (95% CI: 1.89-2.40), respectively, compared with robust participants (both *P* < 0.001) (**Supplementary Table 3**). The metabolic signature also remained associated with MASLD after adjustment for frailty (HR = 1.18, 95% CI: 1.14-1.23 per 1-SD increase; *P* < 0.001) (**Supplementary Table 3**).

### 3.4 Mediation analysis

**Figure 4:**
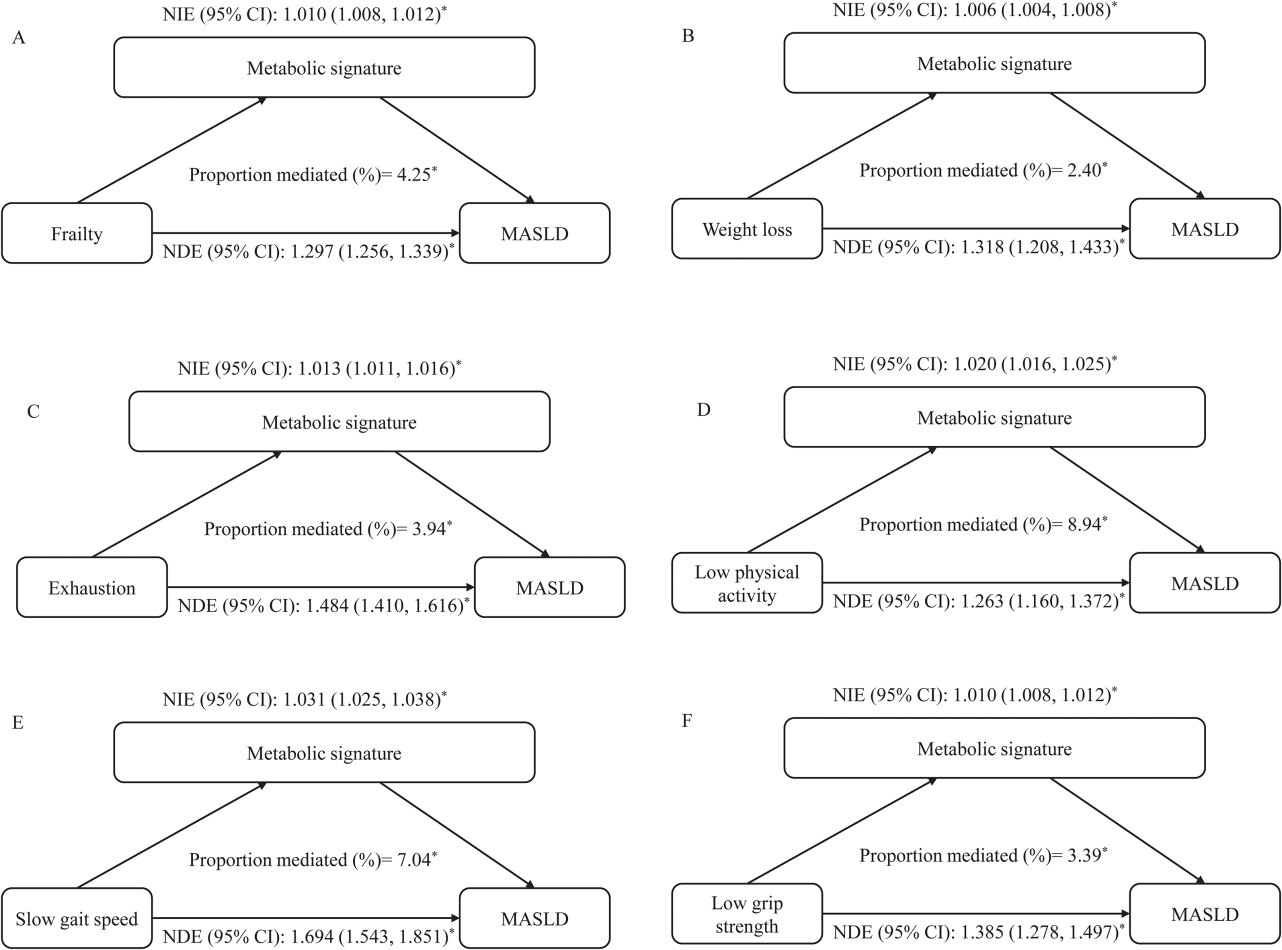
The mediating role of metabolic signature on the association of frailty and its components with incident MASLD. Model adjusted for age, sex, Townsend deprivation index, educational qualifications, smoking status, alcohol use status, dietary pattern, central obesity, high glycaemia/diabetes, high blood pressure/hypertension, low HDL, high triglycerides. **(A)** Mediation analysis for the association between Frailty and MASLD. **(B)** Mediation analysis for the association between Weight loss and MASLD. **(C)** Mediation analysis for the association between Exhaustion and MASLD. **(D)** Mediation analysis for the association between Low physical activity and MASLD. **(E)** Mediation analysis for the association between Slow gait speed and MASLD. **(F)** Mediation analysis for the association between Low grip strength and MASLD. NDE, natural direct effect; NIE, natural indirect effect. The symbol '*' indicates statistical significance with a p-value less than 0.05 (P < 0.05).

Mediation analyses revealed a significant partial mediation effect of the metabolic signature in the associations of frailty and its components with MASLD risk. The metabolic signature significantly mediated the association between frailty and MASLD, accounting for 4.25% of the total effect (**Figure 4A**). The metabolic signature also significantly mediated the associations between individual frailty components and MASLD, with the proportion mediated ranging from 2.40% (unintentional weight loss) to 8.94% (low physical activity) (**Figures 4B–F**).

## 4 Discussion

In this large prospective study of 244,187 UK Biobank participants, we provided a comprehensive investigation of the interrelationships among physical frailty, its individual components, a frailty-related metabolic signature, and incident MASLD. Our findings demonstrate that frailty status, individual frailty components, and the metabolic signature were each independently associated with MASLD risk, with the metabolic signature significantly mediating 4.25% of the frailty-MASLD association, suggesting a modest but significant metabolic pathway linking physical frailty to hepatic steatosis.

To our knowledge, this is the first study to investigate the relationship between frailty-related metabolic signatures and incident MASLD. We first identified frailty-associated metabolites and then constructed a composite metabolic signature, demonstrating its prospective association with MASLD risk and its mediating role in the frailty-MASLD relationship. Previous research has established associations between frailty and various blood-based metabolites (28, 29). For instance, frailty has been linked to alterations in lipoproteins (VLDL, HDL) and amino acids (leucine, lysine) (28), while physical inactivity affects glycogenic amino acid pathways (29). Our findings corroborate and extend these observations by demonstrating that frailty influences a broad spectrum of metabolites, including lipids, lipoprotein subclasses, fatty acids, amino acids, ketone bodies, and inflammation-related markers. Prior studies have also reported associations between specific metabolites and MASLD risk (30, 31). However, no research has integrated frailty, metabolic signatures, and MASLD into a unified prospective analysis.

Few studies have directly evaluated the association between physical frailty and MASLD. However, frailty has been associated with liver cancer (18), diabetes mellitus (32) (a well-established risk factor for MASLD), and abdominal obesity (33) in prior research. Our findings extend this literature by demonstrating a direct prospective association between frailty and incident MASLD. Furthermore, we suggest that frailty-related metabolic dysregulation represents a potential mechanistic pathway, rather than merely a correlated feature. We observed a significant partial mediation effect, implying that metabolic alterations may serve as an objective, multidimensional biomarker capturing the systemic perturbations associated with frailty while reducing analytical complexity.

The liver plays a central role in endogenous metabolism, including amino acid metabolism, gluconeogenesis, and lipid metabolism (34). We hypothesize a bidirectional physiological crosstalk wherein frailty induces systemic metabolic dysregulation that is characterized by perturbations in amino acid catabolism, lipid mobilization, and inflammatory status (35), which in turn predisposes to hepatic steatosis. Mechanistically, disturbances in lipid metabolism, such as elevated triglycerides and atherogenic lipoprotein profiles (e.g., small dense LDL), enhance hepatic fat accumulation by increasing free fatty acid flux and impairing β-oxidation (36, 37). Concurrently, the chronic low-grade inflammation (inflammaging) observed in frail individuals, marked by elevated pro-inflammatory cytokines (e.g., IL-6, TNF-α) and acute-phase proteins (e.g., CRP) (38, 39), promotes insulin resistance and hepatocellular lipotoxicity, thereby exacerbating MASLD progression (40, 41). Our metabolic signature effectively captures these interconnected biological pathways, serving as a systemic biomarker of the frailty-MASLD axis.

Our findings have important clinical implications. The frailty-related metabolic signature may serve as an objective, multidimensional biomarker for the early detection of MASLD in frail individuals, potentially identifying at-risk patients before the onset of overt symptoms. Currently, MASLD diagnosis relies on imaging or invasive biopsy, methods that are resource-intensive and impractical for population-wide screening. In contrast, a blood-based metabolic signature could enable cost-effective risk stratification in primary care settings. Furthermore, the specific metabolites identified, particularly those involved in lipid metabolism and inflammation, represent potential therapeutic targets. Interventions targeting these shared metabolic pathways, such as omega-3 fatty acid supplementation and anti-inflammatory strategies, may concurrently ameliorate frailty and prevent MASLD progression. These hypotheses warrant validation in future clinical trials.

Our study has several strengths. First, we employed a high-throughput NMR-based metabolomic platform quantifying 251 metabolic biomarkers, enabling comprehensive profiling of lipoprotein metabolism, fatty acid composition, and inflammatory markers. Second, the large sample size (n>240,000) and long follow-up (median 13.7 years) provided robust statistical power to detect modest associations and establish temporal sequences. Finally, the prospective design with incident MASLD ascertainment minimizes reverse causation bias inherent in cross-sectional studies.

This study has several limitations as well. First, four frailty components (weight loss, exhaustion, low physical activity, slow gait speed) were self-reported, introducing potential recall bias and misclassification; objective accelerometry data would strengthen future research. Second, we could not distinguish unintentional from intentional weight loss, potentially diluting the frailty-MASLD association. Third, despite extensive covariate adjustment, residual confounding from unmeasured factors (e.g., genetic predisposition, gut microbiome composition) cannot be excluded. Fourth, participants were predominantly White, limiting generalizability to other ethnic groups with distinct metabolic profiles and MASLD risk factors. Finally, the mediation proportion (4.25%) was modest, suggesting that additional pathways (e.g., mitochondrial dysfunction, autophagy impairment) contribute to the frailty-MASLD relationship and warrant investigation.

## 5 Conclusion

In conclusion, this large prospective study demonstrates that physical frailty is independently associated with incident MASLD and identifies a frailty-related metabolic signature, comprising lipoprotein lipids, fatty acids, and inflammatory markers, that partially mediates this association. These findings elucidate a metabolic pathway linking frailty to hepatic steatosis and highlight the potential of blood-based metabolic profiling for the early detection of MASLD in frail individuals. Future research should prioritize validating this signature across diverse populations and evaluating targeted interventions that address shared metabolic vulnerabilities.

## Data Availability

The data analysed in this study is subject to the following licenses/restrictions: The UK Biobank data are available to approved researchers through a formal application process. Researchers must register, submit a research proposal, and agree to the UK Biobank’s data access policies to access the data. The data are not publicly available without prior approval due to ethical and privacy considerations. Requests to access these datasets should be directed to https://www.ukbiobank.ac.uk/enable-your-research/apply-for-access.

## References

[1] Younossi ZM Golabi P Paik JM Henry A Van Dongen C Henry L . The global epidemiology of nonalcoholic fatty liver disease (Nafld) and nonalcoholic steatohepatitis (Nash): a systematic review. Hepatology. (2023) 77:1335–47. doi: 10.1097/hep.0000000000000004. PMID: 36626630 PMC10026948

[2] Riazi K Azhari H Charette JH Underwood FE King JA Afshar EE . The prevalence and incidence of Nafld worldwide: a systematic review and meta-analysis. Lancet Gastroenterol Hepatol. (2022) 7:851–61. doi: 10.1016/s2468-1253(22)00165-0. PMID: 35798021

[3] Ginès P Serra-Burriel M Kamath PS . Metabolic dysfunction-associated steatotic liver disease-the new epidemic of chronic liver disease. JAMA Netw Open. (2025) 8:e2516381. doi: 10.1001/jamanetworkopen.2025.16381. PMID: 40526389

[4] Perumpail BJ Khan MA Yoo ER Cholankeril G Kim D Ahmed A . Clinical epidemiology and disease burden of nonalcoholic fatty liver disease. World J Gastroenterol. (2017) 23:8263–76. doi: 10.3748/wjg.v23.i47.8263. PMID: 29307986 PMC5743497

[5] Witkowski M Moreno SI Fernandes J Johansen P Augusto M Nair S . The economic burden of non-alcoholic steatohepatitis: a systematic review. Pharmacoeconomics. (2022) 40:751–67. doi: 10.1007/s40273-022-01140-y. PMID: 35789987 PMC9300564

[6] Kotronen A Yki-Järvinen H . Fatty liver: a novel component of the metabolic syndrome. Arterioscler Thromb Vasc Biol. (2008) 28:27–38. doi: 10.1161/atvbaha.107.147538. PMID: 17690317

[7] Younossi ZM . Non-alcoholic fatty liver disease - a global public health perspective. J Hepatol. (2019) 70:531–44. doi: 10.1016/j.jhep.2018.10.033. PMID: 30414863

[8] Dent E Martin FC Bergman H Woo J Romero-Ortuno R Walston JD . Management of frailty: opportunities, challenges, and future directions. Lancet. (2019) 394:1376–86. doi: 10.1016/s0140-6736(19)31785-4. PMID: 31609229

[9] Hanlon P Nicholl BI Jani BD Lee D McQueenie R Mair FS . Frailty and pre-frailty in middle-aged and older adults and its association with multimorbidity and mortality: a prospective analysis of 493 737 Uk Biobank participants. Lancet Public Health. (2018) 3:e323-e32. doi: 10.1016/s2468-2667(18)30091-4. PMID: 29908859 PMC6028743

[10] Zhong Q Zhou R Huang YN Huang RD Li FR Chen HW . Frailty and risk of metabolic dysfunction-associated steatotic liver disease and other chronic liver diseases. J Hepatol. (2025) 82:427–37. doi: 10.1016/j.jhep.2024.08.024. PMID: 39218228

[11] Mocciaro G Allison M Jenkins B Azzu V Huang-Doran I Herrera-Marcos LV . Non-alcoholic fatty liver disease is characterised by a reduced polyunsaturated fatty acid transport via free fatty acids and high-density lipoproteins (Hdl). Mol Metab. (2023) 73:101728. doi: 10.1016/j.molmet.2023.101728. PMID: 37084865 PMC10176260

[12] Rada P González-Rodríguez Á García-Monzón C Valverde ÁM . Understanding lipotoxicity in Nafld pathogenesis: is Cd36 a key driver? Cell Death Dis. (2020) 11:802. doi: 10.1038/s41419-020-03003-w. PMID: 32978374 PMC7519685

[13] Bar N Korem T Weissbrod O Zeevi D Rothschild D Leviatan S . A reference map of potential determinants for the human serum metabolome. Nature. (2020) 588:135–40. doi: 10.1038/s41586-020-2896-2. PMID: 33177712

[14] Ran S Zhang J Tian F Qian ZM Wei S Wang Y . Association of metabolic signatures of air pollution with Masld: observational and Mendelian randomization study. J Hepatol. (2025) 82:560–70. doi: 10.1016/j.jhep.2024.09.033. PMID: 39349253

[15] Tian F Wang Y Qian ZM Ran S Zhang Z Wang C . Plasma metabolomic signature of healthy lifestyle, structural brain reserve and risk of dementia. Brain. (2025) 148:143–53. doi: 10.1093/brain/awae257. PMID: 39324695

[16] Collins R . What makes Uk Biobank special? Lancet. (2012) 379:1173–4. doi: 10.1016/s0140-6736(12)60404-8. PMID: 22463865

[17] Fried LP Tangen CM Walston J Newman AB Hirsch C Gottdiener J . Frailty in older adults: evidence for a phenotype. J Gerontol A Biol Sci Med Sci. (2001) 56:M146–56. doi: 10.1093/gerona/56.3.m146. PMID: 11253156

[18] Liu F Peng Y Wang P Qiao Y Si C Wang X . Associations of physical frailty with incidence and mortality of overall and site-specific cancers: a prospective cohort study from Uk Biobank. Prev Med. (2023) 177:107742. doi: 10.1016/j.ypmed.2023.107742. PMID: 37866694

[19] Julkunen H Cichońska A Slagboom PE Würtz P . Metabolic biomarker profiling for identification of susceptibility to severe pneumonia and Covid-19 in the general population. Elife. (2021) 10. doi: 10.7554/eLife.63033. PMID: 33942721 PMC8172246

[20] Ritchie SC Surendran P Karthikeyan S Lambert SA Bolton T Pennells L . Quality control and removal of technical variation of Nmr metabolic biomarker data in ~120,000 Uk Biobank participants. Sci Data. (2023) 10:64. doi: 10.1038/s41597-023-01949-y. PMID: 36720882 PMC9887579

[21] Würtz P Kangas AJ Soininen P Lawlor DA Davey Smith G Ala-Korpela M . Quantitative serum nuclear magnetic resonance metabolomics in large-scale epidemiology: a primer on -omic technologies. Am J Epidemiol. (2017) 186:1084–96. doi: 10.1093/aje/kwx016. PMID: 29106475 PMC5860146

[22] Rinella ME Sookoian S . From Nafld to Masld: updated naming and diagnosis criteria for fatty liver disease. J Lipid Res. (2024) 65:100485. doi: 10.1016/j.jlr.2023.100485. PMID: 38103785 PMC10824973

[23] Mozaffarian D . Dietary and policy priorities for cardiovascular disease, diabetes, and obesity: a comprehensive review. Circulation. (2016) 133:187–225. doi: 10.1161/circulationaha.115.018585. PMID: 26746178 PMC4814348

[24] Huang PL . A comprehensive definition for metabolic syndrome. Dis Model Mech. (2009) 2:231–7. doi: 10.1242/dmm.001180. PMID: 19407331 PMC2675814

[25] Kassi E Pervanidou P Kaltsas G Chrousos G . Metabolic syndrome: definitions and controversies. BMC Med. (2011) 9:48. doi: 10.1186/1741-7015-9-48. PMID: 21542944 PMC3115896

[26] Valeri L Vanderweele TJ . Mediation analysis allowing for exposure-mediator interactions and causal interpretation: theoretical assumptions and implementation with Sas and Spss macros. Psychol Methods. (2013) 18:137–50. doi: 10.1037/a0031034. PMID: 23379553 PMC3659198

[27] Shi B Choirat C Coull BA VanderWeele TJ Valeri L . Cmaverse: a suite of functions for reproducible causal mediation analyses. Epidemiology. (2021) 32:e20-e2. doi: 10.1097/ede.0000000000001378. PMID: 34028370

[28] Pu Y Sun Z Zhang H Huang Q Wang Z Mei Z . Gut microbial features and circulating metabolomic signatures of frailty in older adults. Nat Aging. (2024) 4:1249–62. doi: 10.1038/s43587-024-00678-0. PMID: 39054372

[29] Sahu B Pani S Swalsingh G Senapati U Pani P Pati B . Long-term physical inactivity induces significant changes in biochemical pathways related to metabolism of proteins and glycerophospholipids in mice. Mol Omics. (2024) 20:64–77. doi: 10.1039/d3mo00127j. PMID: 37909389

[30] Hu C Wang T Zhuang X Sun Q Wang X Lin H . Metabolic analysis of early nonalcoholic fatty liver disease in humans using liquid chromatography-mass spectrometry. J Transl Med. (2021) 19:152. doi: 10.1186/s12967-021-02820-7. PMID: 33858428 PMC8050915

[31] Caussy C Ajmera VH Puri P Hsu CL Bassirian S Mgdsyan M . Serum metabolites detect the presence of advanced fibrosis in derivation and validation cohorts of patients with non-alcoholic fatty liver disease. Gut. (2019) 68:1884–92. doi: 10.1136/gutjnl-2018-317584. PMID: 30567742 PMC8328048

[32] Kelly SG Wu K Tassiopoulos K Erlandson KM Koletar SL Palella FJ . Frailty is an independent risk factor for mortality, cardiovascular disease, bone disease, and diabetes among aging adults with human immunodeficiency virus. Clin Infect Dis. (2019) 69:1370–6. doi: 10.1093/cid/ciy1101. PMID: 30590451 PMC6938206

[33] Afonso C Sousa-Santos AR Santos A Borges N Padrão P Moreira P . Frailty status is related to general and abdominal obesity in older adults. Nutr Res. (2021) 85:21–30. doi: 10.1016/j.nutres.2020.10.009. PMID: 33422742

[34] Ma X Huang T Chen X Li Q Liao M Fu L . Molecular mechanisms in liver repair and regeneration: from physiology to therapeutics. Signal Transduct Target Ther. (2025) 10:63. doi: 10.1038/s41392-024-02104-8. PMID: 39920130 PMC11806117

[35] Abdullah G Akpan A Phelan MM Wright HL . New insights into healthy ageing, inflammageing and frailty using metabolomics. Front Aging. (2024) 5:1426436. doi: 10.3389/fragi.2024.1426436. PMID: 39044748 PMC11263002

[36] Karakousis ND Chrysavgis L Chatzigeorgiou A Papatheodoridis G Cholongitas E . Frailty in metabolic syndrome, focusing on nonalcoholic fatty liver disease. Ann Gastroenterol. (2022) 35:234–42. doi: 10.20524/aog.2022.0705. PMID: 35599934 PMC9062844

[37] Badmus OO Hillhouse SA Anderson CD Hinds TD Stec DE . Molecular mechanisms of metabolic associated fatty liver disease (Mafld): functional analysis of lipid metabolism pathways. Clin Sci (Lond). (2022) 136:1347–66. doi: 10.1042/cs20220572. PMID: 36148775 PMC9508552

[38] Pansarasa O Mimmi MC Davin A Giannini M Guaita A Cereda C . Inflammation and cell-to-cell communication, two related aspects in frailty. Immun Ageing. (2022) 19:49. doi: 10.1186/s12979-022-00306-8. PMID: 36289502 PMC9598012

[39] Xu Y Li Q Jiao X . Immunosenescence and metabolic reprogramming in Masld: an age-dependent immunometabolic vicious cycle and therapeutic opportunities. Front Cell Dev Biol. (2025) 13:1650677. doi: 10.3389/fcell.2025.1650677. PMID: 41133219 PMC12540461

[40] Marcos-Pérez D Sánchez-Flores M Proietti S Bonassi S Costa S Teixeira JP . Association of inflammatory mediators with frailty status in older adults: results from a systematic review and meta-analysis. Geroscience. (2020) 42:1451–73. doi: 10.1007/s11357-020-00247-4. PMID: 32803650 PMC7732924

[41] Qiu X Shen S Jiang N Feng Y Yang G Lu D . Associations between systemic inflammatory biomarkers and metabolic dysfunction associated steatotic liver disease: a cross-sectional study of Nhanes 2017-2020. BMC Gastroenterol. (2025) 25:42. doi: 10.1186/s12876-025-03625-4. PMID: 39881239 PMC11776320

