# Supplementary material for: Metabolomic signatures mediate the association between physical frailty and metabolic dysfunction-associated steatotic liver disease: a prospective cohort study
Source: Front Endocrinol (Lausanne). 2026 May 12;17:1845611. doi: 10.3389/fendo.2026.1845611 (PMC13201151; doi:10.3389/fendo.2026.1845611)
Supplement: Supplementary file 1 [file DataSheet1.docx]

Supplementary Material

# Supplementary Figures and Tables

## Supplementary Figures


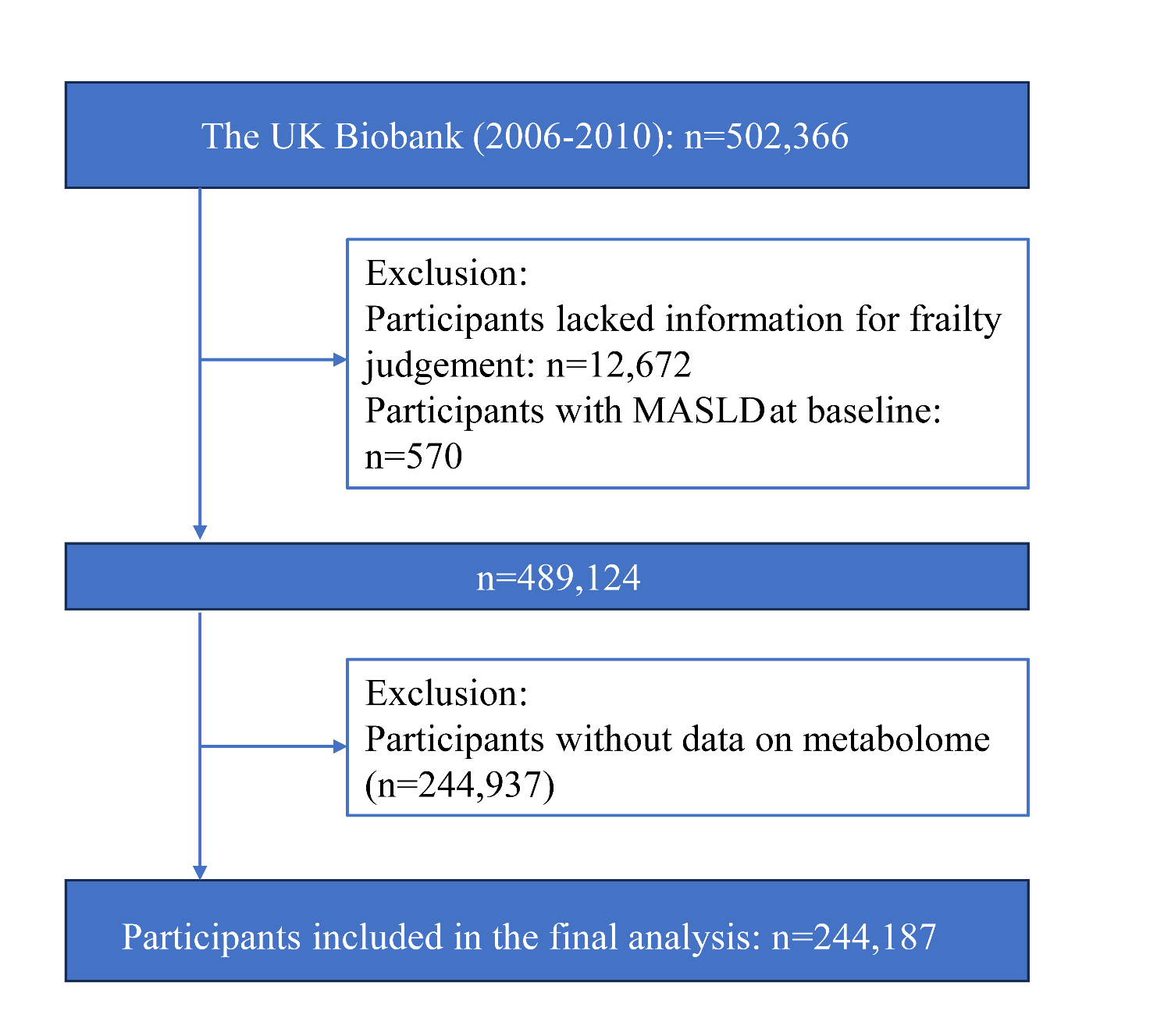


**Supplementary** **Figure 1** Diagram of participants included in the analyses. MASLD: non-alcoholic fatty liver diseases.


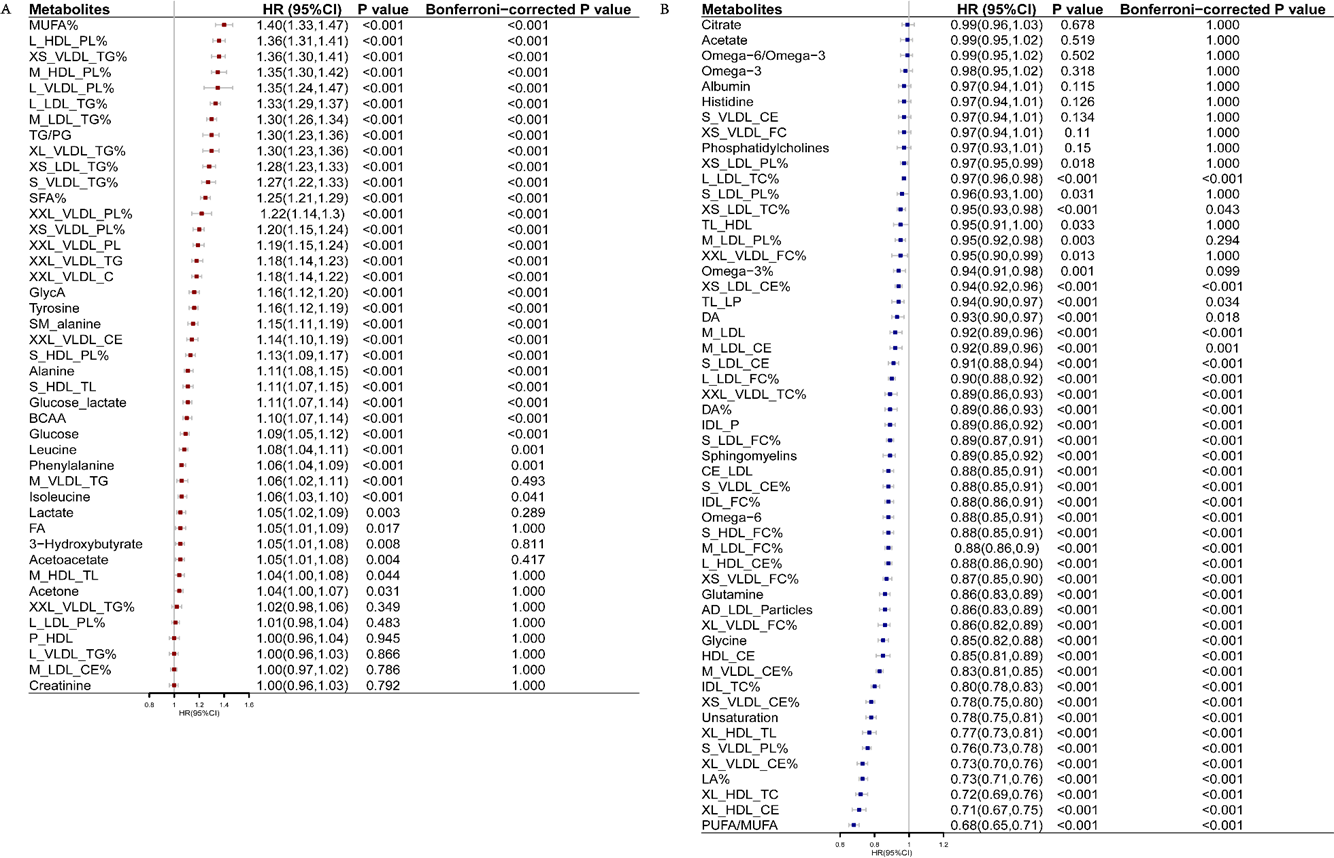


**Supplementary** **Figure 2 Association between 96 metabolites and incident MASLD. (A) positive association, (B) negative association.** Abbreviation: AD, average diameter; CE, cholesteryl ester; CI, confidence interval; DA, docosahexaenoic acid; FA, total fatty acids; FC, free cholesterol; GlycA, glycoprotein acetyls; HDL, high-density lipoprotein; HR, hazard ratio;IDL, intermediate density lipoprotein; L, large; LA, linoleic acid; LDL, low-density lipoprotein; LP, lipoprotein particles; M, medium; MUFA, monounsaturated fatty acids; PG, phosphoglycerides; PL, phospholipid; PUFA, polyunsaturated fatty acids; S, small; SFA, saturated fatty acids; SM, Spectrometer; TC, total cholesterol; TL, total lipids; TP, total phospholipids; VLDL, very low-density lipoprotein; XL, very large; XS, very small; XXL, especially large.

## Supplementary Tables

**Supplementary** **Table 1 Frailty definition and cut-off points in UK Biobank.**

| Individual components | Criteria | Field IDs |
| --- | --- | --- |
| Weight loss | Self-reported: “Compared with one year ago, has your weight changed?”  Response: yes, lost weight = 1; other = 0;  Do not know/Prefer not to answer = missing data. | 2306 |
| Exhaustion | Self-reported: “Over the past two weeks, how often have you felt tired or had little energy?”  Response: more than half the days or nearly every day = 1; other = 0;  Do not know/Prefer not to answer = missing data. | 2080 |
| Low physical activity | Quintiles of sex- and age-specific levels of total MET minutes per week derived from IPAQ.  The lowest 20% of total MET minutes per week = 1; other = 0;  No response/ Prefer not to answer = missing data. | 31, 21022, 22037, 22038, 22039 |
| Slow gait speed | Self-reported: “How would you describe your usual walking pace?”  Response: slow = 1; other = 0;  Do not know/Prefer not to answer = missing data. | 924 |
| Low grip strength | Measured grip strength expressed in kg by sex- and BMI- adjusted cut-off points.  Cut-off points:  Male  If BMI ≤24.0 kg/m2 & grip strength ≤29 kg;  If BMI 24.1 to 26.0 kg/m2 & grip strength ≤30 kg;  If BMI 26.1 to 28.0 kg/m2 & grip strength ≤30 kg;  If BMI >28.0 kg/m2 & grip strength ≤32 kg Female  If BMI ≤23.0 kg/m2 & grip strength ≤17 kg;  If BMI 23.1 to 26.0 kg/m2 & grip strength ≤17.3 kg;  If BMI 26.1 to 29.0 kg/m2 & grip strength ≤18 kg;  If BMI >29.0 kg/m2 & grip strength ≤21 kg;  If data on BMI or grip strength is not available = missing data. | 31, 21001, 46, 47 |

Five individual components used for definition of physical frailty phenotypes are shown.

Abbreviations: BMI, body mass index; IPAQ, International Physical Activity Questionnaire; MET, metabolic equivalent.

**Supplementary** **Table 2 Definitions of cardiometabolic risk factors for MASLD diagnosis and corresponding UK Biobank field IDs.**

| Cardiometabolic risk factor | Criteria | Field IDs |
| --- | --- | --- |
| Overweight/obesity | BMI ≥ 25 kg/m² (≥ 23 kg/m² for Asian populations) or elevated waist circumference | 21001; 48 |
| Type 2 diabetes mellitus or impaired glucose regulation | Fasting glucose ≥ 100 mg/dL (≥ 5.6 mmol/L), 2h OGTT ≥ 140 mg/dL, HbA1c ≥ 5.7%, or use of glucose-lowering medications | 30740; 30750; 2443; 20003; 6153/6177 |
| Hypertension | Blood pressure ≥ 130/85 mmHg, or use of antihypertensive medications | 4080; 4079; 6150; 6153/6177 |
| Hypertriglyceridemia | TG ≥ 150 mg/dL (≥ 1.70 mmol/L), or use of lipid-lowering medications | 30870; 6153/6177 |
| Low HDL-cholesterol | HDL-C < 40 mg/dL (men) or < 50 mg/dL (women), or use of lipid-lowering medications | 30760; 6153/6177 |

**Supplementary** **Table 3 Association between frailty status and metabolic signature with the risk of incident MASLD.**

|  | **Events/ Total participants** | **Model1** | | **Model2** | | **Model3** | |
| --- | --- | --- | --- | --- | --- | --- | --- |
|  |  | **HR (95%CI)** | ***p* value** | **HR (95%CI)** | ***p* value** | **HR (95%CI)** | ***p* value** |
| **Frailty status** | | | | | | | |
| Non-frailty | 1,148/ 126,115 | Reference |  | Reference |  | Reference |  |
| Pre-frailty | 1,838/ 106,873 | 1.96(1.82, 2.11) | < 0.001 | 1.51(1.40, 1.63) | < 0.001 | 1.49(1.38, 1.61) | < 0.001 |
| Frailty | 422/ 11,199 | 4.72(4.22, 5.28) | < 0.001 | 2.22(1.97, 2.50) | < 0.001 | 2.13(1.89, 2.40) | < 0.001 |
| P value for trend |  |  | < 0.001 |  |  |  |  |
| Continuous, per 1-point increase | 3,408/ 244,187 | 1.62(1.57, 1.67) | < 0.001 | 1.30(1.26, 1.34) | < 0.001 | 1.28(1.24, 1.33) | < 0.001 |
| **Metabolic signature** | | | | | | | |
| Q1 | 446/ 61,047 | Reference |  | Reference |  | Reference |  |
| Q2 | 605/ 61,047 | 1.38(1.22, 1.56) | < 0.001 | 1.10(0.97, 1.24) | 0.137 | 1.09(0.97, 1.24) | 0.158 |
| Q3 | 836/ 61,046 | 1.94(1.73, 2.17) | < 0.001 | 1.24(1.11, 1.40) | 0.001 | 1.23(1.10, 1.39) | < 0.001 |
| Q4 | 1,521/ 61,047 | 3.65(3.28, 4.06) | < 0.001 | 1.51(1.35, 1.70) | < 0.001 | 1.47(1.30, 1.65) | < 0.001 |
| Continuous, per 1-point increase | 3,408/ 244,187 | 1.60(1.55, 1.64) | < 0.001 | 1.21(1.16, 1.25) | < 0.001 | 1.18(1.14, 1.23) | < 0.001 |

HR, hazard ratio; MASLD, nonalcoholic fatty liver disease.

Associations of frailty status and metabolic signature with risk of incident MASLD were analysis by using Cox proportional hazard models. Model 1 was adjusted for age and sex. Model 2 was adjusted for Townsend deprivation index, educational qualifications, smoking status, alcohol use status, dietary pattern, central obesity, high glycaemia/diabetes, high blood pressure/hypertension, low HDL, high triglycerides. Model 3 was a multivariable model that incorporated both frailty status and metabolic signature for adjustment, was used to explore the independent role of frailty status and metabolic signature on MASLD. p values <0.05 were considered statistically significant.

**Supplementary** **Table 4** Association between frailty status and the risk of incident MASLD stratified by major covariates.

| Subgroup | Non frailty | Pre frailty | | Frailty | | p value for interaction |
| --- | --- | --- | --- | --- | --- | --- |
|  | HR (95%CI) | HR (95%CI) | p value | HR (95%CI) | p value |  |
| **Sex** |  |  |  |  |  | 0.344 |
| Male | 1.00 (reference) | 1.54(1.39, 1.71) | <0.001 | 2.09(1.74, 2.51) | <0.001 |  |
| Female | 1.00 (reference) | 1.44(1.30, 1.61) | <0.001 | 2.14(1.83, 2.51) | <0.001 |  |
| **Age** |  |  |  |  |  | 0.060 |
| <60 | 1.00 (reference) | 1.61(1.46, 1.78) | <0.001 | 2.32(1.97, 2.73) | <0.001 |  |
| ≥60 | 1.00 (reference) | 1.35(1.20, 1.50) | <0.001 | 1.91(1.60, 2.27) | <0.001 |  |
| **Smoking** |  |  |  |  |  | 0.153 |
| Never | 1.00 (reference) | 1.51(1.35, 1.69) | <0.001 | 2.19(1.82, 2.64) | <0.001 |  |
| Former/current | 1.00 (reference) | 1.48(1.33, 1.63) | <0.001 | 2.12(1.81, 2.48) | <0.001 |  |
| **Drinking** |  |  |  |  |  | 0.397 |
| Never | 1.00 (reference) | 1.74(1.20, 2.54) | 0.005 | 2.38(1.50, 3.76) | <0.001 |  |
| Former/current | 1.00 (reference) | 1.49(1.38, 1.61) | <0.001 | 2.19(1.94, 2.48) | <0.001 |  |
| **Central obesity** |  |  |  |  |  | 0.024 |
| Yes | 1.00 (reference) | 1.4(1.27, 1.54) | <0.001 | 2.01(1.75, 2.31) | <0.001 |  |
| No | 1.00 (reference) | 1.64(1.45, 1.85) | <0.001 | 2.42(1.91, 3.08) | <0.001 |  |
| **Hypertension** |  |  |  |  |  | 0.891 |
| Yes | 1.00 (reference) | 1.51(1.38, 1.64) | <0.001 | 2.18(1.90, 2.50) | <0.001 |  |
| No | 1.00 (reference) | 1.43(1.21, 1.68) | <0.001 | 1.93(1.51, 2.48) | <0.001 |  |
| **High triglycerides** |  |  |  |  |  | 0.070 |
| Yes | 1.00 (reference) | 1.53(1.39, 1.68) | <0.001 | 2.03(1.75, 2.36) | <0.001 |  |
| No | 1.00 (reference) | 1.41(1.25, 1.59) | <0.001 | 2.28(1.87, 2.78) | <0.001 |  |
| **Low HDL** |  |  |  |  |  | 0.001 |
| Yes | 1.00 (reference) | 1.44(1.27, 1.63) | <0.001 | 1.78(1.49, 2.14) | <0.001 |  |
| No | 1.00 (reference) | 1.5(1.36, 1.65) | <0.001 | 2.5(2.14, 2.92) | <0.001 |  |
| **Diabetes** |  |  |  |  |  | 0.476 |
| Yes | 1.00 (reference) | 1.52(1.32, 1.74) | <0.001 | 2.08(1.72, 2.53) | <0.001 |  |
| No | 1.00 (reference) | 1.47(1.35, 1.61) | <0.001 | 2.17(1.86, 2.53) | <0.001 |  |

Obtained by using multivariable Cox regression model.

**Supplementary** **Table 5** Association between frailty status and metabolic signature with the risk of incident MASLD after excluding incident cases within the first 2 year of follow-up.

|  | Model1 | | Model2 | | Model3 | |
| --- | --- | --- | --- | --- | --- | --- |
|  | HR (95%CI) | *p* value | HR (95%CI) | *p* value | HR (95%CI) | *p* value |
| **Frailty status** |  |  |  |  |  |  |
| Non-frailty | Reference |  | Reference |  | Reference |  |
| Pre-frailty | 1.96(1.81, 2.11) | < 0.001 | 1.51(1.40, 1.63) | < 0.001 | 1.49(1.38, 1.61) | < 0.001 |
| Frailty | 4.69(4.19, 5.26) | < 0.001 | 2.21(1.96, 2.50) | < 0.001 | 2.12(1.88, 2.40) | < 0.001 |
| P value for trend |  | < 0.001 |  |  |  |  |
| Continuous, per 1-point increase | 1.61(1.57, 1.66) | < 0.001 | 1.30(1.26, 1.34) | < 0.001 | 1.28(1.24, 1.32) | < 0.001 |
| **Metabolic signature** |  |  |  |  |  |  |
| Q1 | Reference |  | Reference |  | Reference |  |
| Q2 | 1.40(1.24, 1.59) | < 0.001 | 1.11(0.98, 1.26) | 0.092 | 1.11(0.98, 1.26) | 0.108 |
| Q3 | 1.97(1.75, 2.21) | < 0.001 | 1.27(1.13, 1.43) | < 0.001 | 1.26(1.12, 1.42) | 0.001 |
| Q4 | 3.66(3.29, 4.09) | < 0.001 | 1.54(1.36, 1.73) | < 0.001 | 1.49(1.32, 1.68) | < 0.001 |
| Continuous, per 1-point increase | 1.59(1.55, 1.63) | < 0.001 | 1.20(1.16, 1.25) | < 0.001 | 1.18(1.14, 1.23) | < 0.001 |

HR, hazard ratio; MASLD, nonalcoholic fatty liver disease.

Associations of frailty status and metabolic signature with risk of incident MASLD were analysis by using Cox proportional hazard models. Model 1 was adjusted for age and sex. Model 2 was adjusted for Townsend deprivation index, educational qualifications, smoking status, alcohol use status, dietary pattern, central obesity, high glycaemia/diabetes, high blood pressure/hypertension, low HDL, high triglycerides. Model 3 was a multivariable model that incorporated both frailty status and metabolic signature for adjustment, was used to explore the independent role of frailty status and metabolic signature on MASLD. p values <0.05 were considered statistically significant.

**Supplementary** **Table 6** Association between frailty status and metabolic signature with the risk of incident MASLD after excluding participants with cardiovascular disease, cancer at baseline.

|  | Model1 | | Model2 | | Model3 | |
| --- | --- | --- | --- | --- | --- | --- |
|  | HR (95%CI) | *p* value | HR (95%CI) | *p* value | HR (95%CI) | *p* value |
| **Frailty status** |  |  |  |  |  |  |
| Non-frailty | Reference |  | Reference |  | Reference |  |
| Pre-frailty | 1.74(1.57, 1.93) | < 0.001 | 1.39(1.25, 1.54) | < 0.001 | 1.37(1.23, 1.53) | < 0.001 |
| Frailty | 4.80(4.01, 5.75) | < 0.001 | 2.38(1.97, 2.88) | < 0.001 | 2.29(1.89, 2.78) | < 0.001 |
| P value for trend |  | < 0.001 |  |  |  |  |
| Continuous, per 1-point increase | 1.59(1.51, 1.67) | < 0.001 | 1.29(1.23, 1.36) | < 0.001 | 1.28(1.21, 1.35) | < 0.001 |
| **Metabolic signature** |  |  |  |  |  |  |
| Q1 | Reference |  | Reference |  | Reference |  |
| Q2 | 1.45(1.23, 1.70) | < 0.001 | 1.14(0.97, 1.34) | 0.118 | 1.13(0.96, 1.33) | 0.134 |
| Q3 | 1.81(1.55, 2.12) | < 0.001 | 1.16(0.99, 1.36) | 0.067 | 1.15(0.98, 1.35) | 0.084 |
| Q4 | 3.35(2.89, 3.89) | < 0.001 | 1.46(1.24, 1.72) | < 0.001 | 1.42(1.20, 1.67) | < 0.001 |
| Continuous, per 1-point increase | 1.61(1.55, 1.68) | < 0.001 | 1.21(1.14, 1.29) | < 0.001 | 1.19(1.12, 1.26) | < 0.001 |

HR, hazard ratio; MASLD, nonalcoholic fatty liver disease.

Associations of frailty status and metabolic signature with risk of incident MASLD were analysis by using Cox proportional hazard models. Model 1 was adjusted for age and sex. Model 2 was adjusted for Townsend deprivation index, educational qualifications, smoking status, alcohol use status, dietary pattern, central obesity, high glycaemia/diabetes, high blood pressure/hypertension, low HDL, high triglycerides. Model 3 was a multivariable model that incorporated both frailty status and metabolic signature for adjustment, was used to explore the independent role of frailty status and metabolic signature on MASLD. p values <0.05 were considered statistically significant.

**Supplementary** **Table 7** Association between frailty status and metabolic signature with the risk of incident MASLD using alcohol using frequency instead of alcohol using status.

|  | Model1 | | Model2 | | Model3 | |
| --- | --- | --- | --- | --- | --- | --- |
|  | HR (95%CI) | *p* value | HR (95%CI) | *p* value | HR (95%CI) | *p* value |
| **Frailty status** |  |  |  |  |  |  |
| Non-frailty | Reference |  | Reference |  | Reference |  |
| Pre-frailty | 1.96(1.82, 2.11) | < 0.001 | 1.51(1.40, 1.62) | < 0.001 | 1.49(1.38, 1.60) | < 0.001 |
| Frailty | 4.72(4.22, 5.28) | < 0.001 | 2.24(1.99, 2.52) | < 0.001 | 2.15(1.91, 2.42) | < 0.001 |
| P value for trend |  | < 0.001 |  |  |  |  |
| Continuous, per 1-point increase | 1.62(1.57, 1.67) | < 0.001 | 1.30(1.26, 1.35) | < 0.001 | 1.29(1.25, 1.33) | < 0.001 |
| **Metabolic signature** |  |  |  |  |  |  |
| Q1 | Reference |  | Reference |  | Reference |  |
| Q2 | 1.38(1.22, 1.56) | < 0.001 | 1.10(0.97, 1.24) | 0.135 | 1.09(0.97, 1.24) | 0.158 |
| Q3 | 1.94(1.73, 2.17) | < 0.001 | 1.24(1.11, 1.40) | 0.001 | 1.23(1.09, 1.38) | 0.001 |
| Q4 | 3.65(3.28, 4.06) | < 0.001 | 1.51(1.34, 1.70) | < 0.001 | 1.47(1.30, 1.65) | < 0.001 |
| Continuous, per 1-point increase | 1.60(1.56, 1.64) | < 0.001 | 1.21(1.16, 1.25) | < 0.001 | 1.18(1.14, 1.23) | < 0.001 |

HR, hazard ratio; MASLD, nonalcoholic fatty liver disease.

Associations of frailty status and metabolic signature with risk of incident MASLD were analysis by using Cox proportional hazard models. Model 1 was adjusted for age and sex. Model 2 was adjusted for Townsend deprivation index, educational qualifications, smoking status, alcohol consumption frequency, dietary pattern, central obesity, high glycaemia/diabetes, high blood pressure/hypertension, low HDL, high triglycerides. Model 3 was a multivariable model that incorporated both frailty status and metabolic signature for adjustment, was used to explore the independent role of frailty status and metabolic signature on MASLD. p values <0.05 were considered statistically significant.

**Supplementary** **Table 8** Association between frailty status and metabolic signature with the risk of incident MASLD among participants with complete covariates.

|  | Model1 | | Model2 | | Model3 | |
| --- | --- | --- | --- | --- | --- | --- |
|  | HR (95%CI) | *p* value | HR (95%CI) | *p* value | HR (95%CI) | *p* value |
| **Frailty status** |  |  |  |  |  |  |
| Non-frailty | Reference |  | Reference |  | Reference |  |
| Pre-frailty | 1.97(1.81, 2.13) | < 0.001 | 1.51(1.39, 1.65) | < 0.001 | 1.50(1.38, 1.63) | < 0.001 |
| Frailty | 4.87(4.30, 5.51) | < 0.001 | 2.26(1.98, 2.58) | < 0.001 | 2.17(1.90, 2.48) | < 0.001 |
| P value for trend |  | < 0.001 |  |  |  |  |
| Continuous, per 1-point increase | 1.62(1.57, 1.68) | < 0.001 | 1.30(1.25, 1.35) | < 0.001 | 1.29(1.24, 1.33) | < 0.001 |
| **Metabolic signature** |  |  |  |  |  |  |
| Q1 | Reference |  | Reference |  | Reference |  |
| Q2 | 1.38(1.20, 1.58) | < 0.001 | 1.09(0.95, 1.24) | 0.233 | 1.08(0.95, 1.24) | 0.256 |
| Q3 | 1.86(1.63, 2.11) | < 0.001 | 1.18(1.04, 1.34) | 0.012 | 1.17(1.03, 1.33) | 0.017 |
| Q4 | 3.56(3.17, 4.00) | < 0.001 | 1.42(1.24, 1.62) | < 0.001 | 1.38(1.21, 1.58) | < 0.001 |
| Continuous, per 1-point increase | 1.61(1.56, 1.65) | < 0.001 | 1.20(1.15, 1.25) | < 0.001 | 1.18(1.13, 1.23) | < 0.001 |

HR, hazard ratio; MASLD, nonalcoholic fatty liver disease.

Associations of frailty status and metabolic signature with risk of incident MASLD were analysis by using Cox proportional hazard models. Model 1 was adjusted for age and sex. Model 2 was adjusted for Townsend deprivation index, educational qualifications, smoking status, alcohol consumption frequency, dietary pattern, central obesity, high glycaemia/diabetes, high blood pressure/hypertension, low HDL, high triglycerides. Model 3 was a multivariable model that incorporated both frailty status and metabolic signature for adjustment, was used to explore the independent role of frailty status and metabolic signature on MASLD. p values <0.05 were considered statistically significant.
